# Mitochondrial base editing: from principle, optimization to application

**DOI:** 10.1186/s13578-025-01351-8

**Published:** 2025-01-24

**Authors:** Jinling Tang, Kunzhao Du

**Affiliations:** 1 Clinical Laboratory Center, The First Affiliated Hospital of Guangxi Medical University, Nanning, 530021 China; 2 Jinshan Hospital Center for Neurosurgery, Jinshan Hospital, Institute for Translational Brain Research, State Key Laboratory of Medical Neurobiology, MOE Frontiers Center for Brain Science, Fudan University, Shanghai, 201508 China

**Keywords:** Mitochondrial DNA, mtDNA base editing, DdCBEs, TALENs, Genetic engineering

## Abstract

In recent years, mitochondrial DNA (mtDNA) base editing systems have emerged as bioengineering tools. DddA-derived cytosine base editors (DdCBEs) have been developed to specifically induce C-to-T conversion in mtDNA by the fusion of sequence-programmable transcription activator-like effector nucleases (TALENs) or zinc-finger nucleases (ZFNs), and split deaminase derived from interbacterial toxins. Similar to DdCBEs, mtDNA adenine base editors have been developed with the ability to introduce targeted A-to-G conversions into human mtDNA. In this review, we summarize the principles of mtDNA base-editing systems and elaborate on the evolution of different platforms of mtDNA base editors, including their deaminase replacement, engineering of DddA_tox_ variants, structure optimization and editing outcomes. Finally, we highlight their applications in animal models and human embroys and discuss the future developmental direction and challenges of mtDNA base editors.

## Introduction

Mitochondria are semi-autonomous organelles that generate energy (ATP) for life activities through aerobic respiration, which is essential for the proper functioning of eukaryotic cells [1–3]. In humans, mtDNA is a circular, double-stranded molecule that typically presents between 100 and 100,000 copies per prototypical cell [4]. Human mtDNA has 16,569 base pairs(bp) and contains 37 genes composed of 13 oxidative phosphorylation-related protein-coding genes, two rRNA motifs (coding 12s rRNA and 16s rRNA), and 22 tRNA [4, 5].

Mitochondrial diseases (MDs) are a subset of genetic disorders of dysfunctional mitochondria, mainly as a result of defects in the respiratory chain that affect ATP production [6]. Inherited or acquired mtDNA mutations are usually associated with a number of human diseases, eventually affecting the central nervous and musculoskeletal system [7–10]. MDs can be caused by pathogenic mutations in the nuclear DNA (nDNA) or mtDNA, and researchers have identified over 100 pathogenic point mutations in human mtDNA [11]. Meanwhlie, previously epidemiology research has shown that the minimum prevalence rate of mtDNA mutations is about one in 5000, which is much higher than that of the nuclear mutations causing MDs [12, 13]. Gene therapy is a promising treatment for gene mutations. Therefore, MDs caused by pathogenic mutations may be solved by gene correction.

In recent years, much advancement has been made in genome editing technology, which allows precise base editing of nuclear genes in cells, tissues, and embryos [14–18]. nDNA base editors rely on cytidine deaminases operating on single-stranded nucleic acids, and researchers usually employ a clustered regularly interspaced short palindromic repeats (CRISPR)-Cas system to unwind double-stranded DNA (dsDNA) [19–22]. However, the delivery of guide RNA (gRNA) into mitochondria is technically challenging [23, 24]. Additionally, mitochondria lack common mechanisms for DNA double-strand break repair pathways, further limiting the application of DNA base editors [23, 24]. Thus, manipulation of mtDNA has long been limited to targeted destruction of the mitochondrial genome by designer nucleases.

**Fig. 1:**
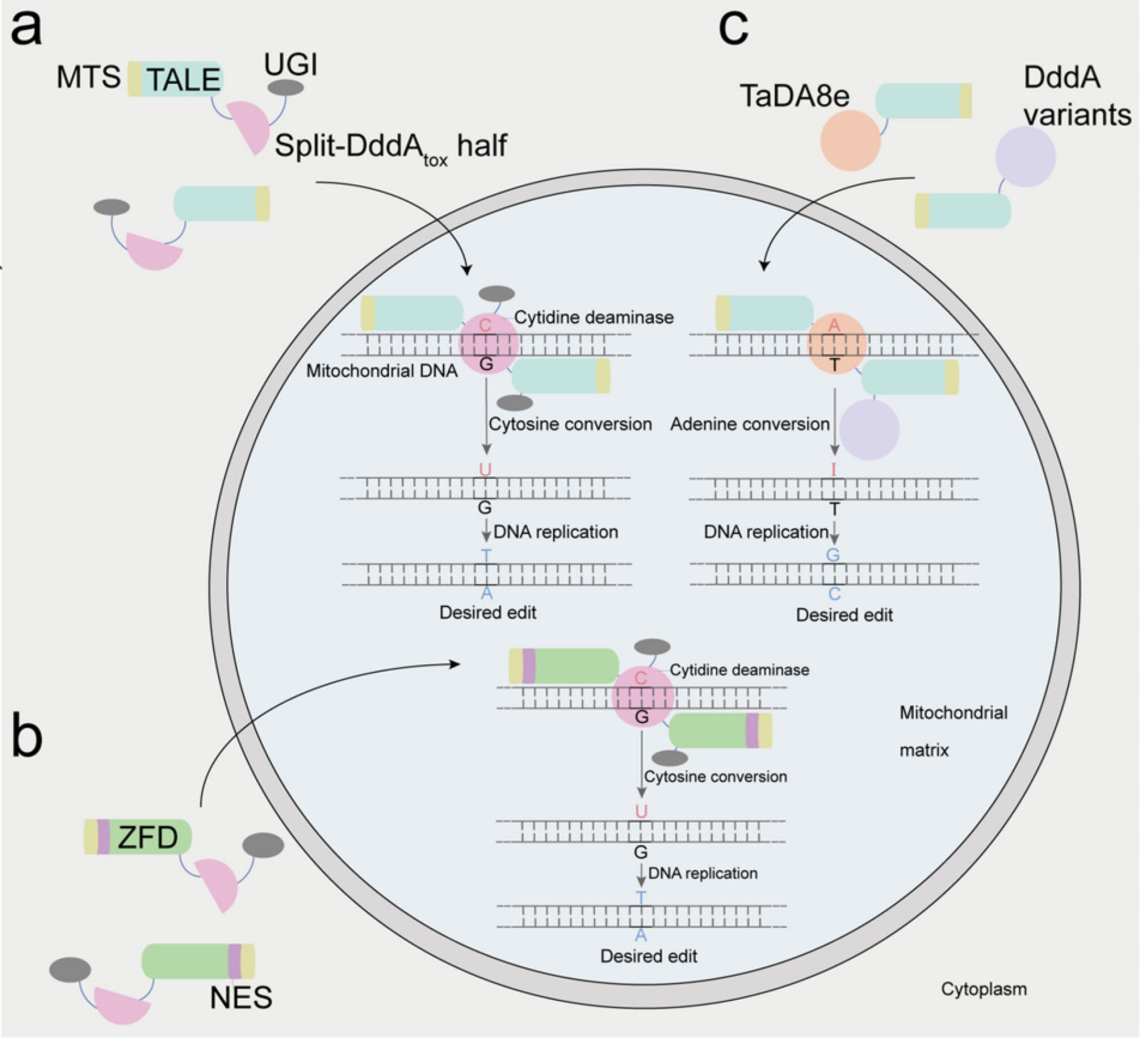
The principle of mtDNA base editing. **(a)** DdCBEs mediate C•G-to-T•A base editing. The MTS is a short signal peptide that guides the transport of DdCBEs protein to the interior of the mitochondria. Transcription activator-like effector (TALE) proteins recognize specific mtDNA sequences, and mediates separation of local mtDNA strands. Two split-DddA_tox_ halves reconstitute deamination activity only when assembled adjacently on target mtDNA, and then mediates cytosine (C) to uracil (U). A tethered UGI can block the base excision to protect U•G intermediate. The resulting U•G heteroduplex can be permanently converted to T•A base pair through DNA replication or DNA repair. **(b)** ZF-based cytidine mtDNA base editors mediate C•G-to-T•A base editing. MTS and NES sequences are linking to the N-terminus of custom-designed zinc finger deaminase (ZFD), and targeted to mitochondrial genes. Two split-DddA_tox_ halves recombine and then mediates cytosine (C) to thymine (T). **(c)** Adenine mtDNA base editor mediated A•T-to-G•C base editing. The editing proteins are guided by MTS into the interior of mitochondria. custom-designed TALE targets specific mtDNA sequences. DddA variants function in cis or in trans with a deoxyadenosine deaminase variant (termed TaDA8e) to catalyze adenine (A) to inosine (I) conversion. The resulting I•T heteroduplex can be permanently converted to a G•C base pair through DNA replication or DNA repair

Recently, several groups have developed alternative mitochondrial genome strategies for precise base editing, such as DdCBEs, mitochondria-targeting zinc finger deaminases (mitoZFDs), and TALE-linked deaminases (TALEDs) [25–27]. These editors can directly convert C•G-to-T•A or A•T-to-G•C at the desired sites in the mtDNA without relying on gRNA. Generally, DdCBEs and mitoZFDs function in mitochondria with two activities: with the guidance of mitochondrial targeting sequence (MTS), DdCBE or mitoZFD protein enters the mitochondria, and the left and right arms bind to each side of the target editing site, thus forming a complete DddA_tox_ protein to catalyze base conversion (Fig. 1ab) [25, 26]. Likewise, TALEDs protein enters the mitochondria under the guidance of MTS. Adenine deaminase TadA8e is responsible for catalyzing base conversion in mtDNA (Fig. 1c) [27, 28]. Therefore, the advent of this technology holds promise for treating mitochondrial diseases.

In this review, we focus on the design and optimization of mtDNA base-editing platforms, including deaminase replacement, engineering of DddA_tox_ variants, structure optimization and editing outcomes. Here, we highlight the applications of mtDNA base editing in mouse models and human embryos. In addition, we discuss the challenges and prospects of mtDNA base editors. Base editors are becoming effective mtDNA base editing tools for establishing novel disease models, offering a new possibility for personalized gene therapies for the treatment of mitochondrial dysfunction.

## Creation and optimization of base editors of C•G-to-T•A in mitochondrial DNA

### TALE-based cytidine mtDNA base editors

The establishment of canonical DdCBEs are based on TALENs. Studies showed that canonical DdCBEs are generally composed of DddA_tox_ (the deaminase domain of DddA) halves, transcription activator-like effector (TALE) proteins, a MTS, and an uracil glycosylase inhibitor (UGI) (Fig. 1a), enabling direct C•G-to-T•A conversions in human mtDNA [25].

**Fig. 2:**
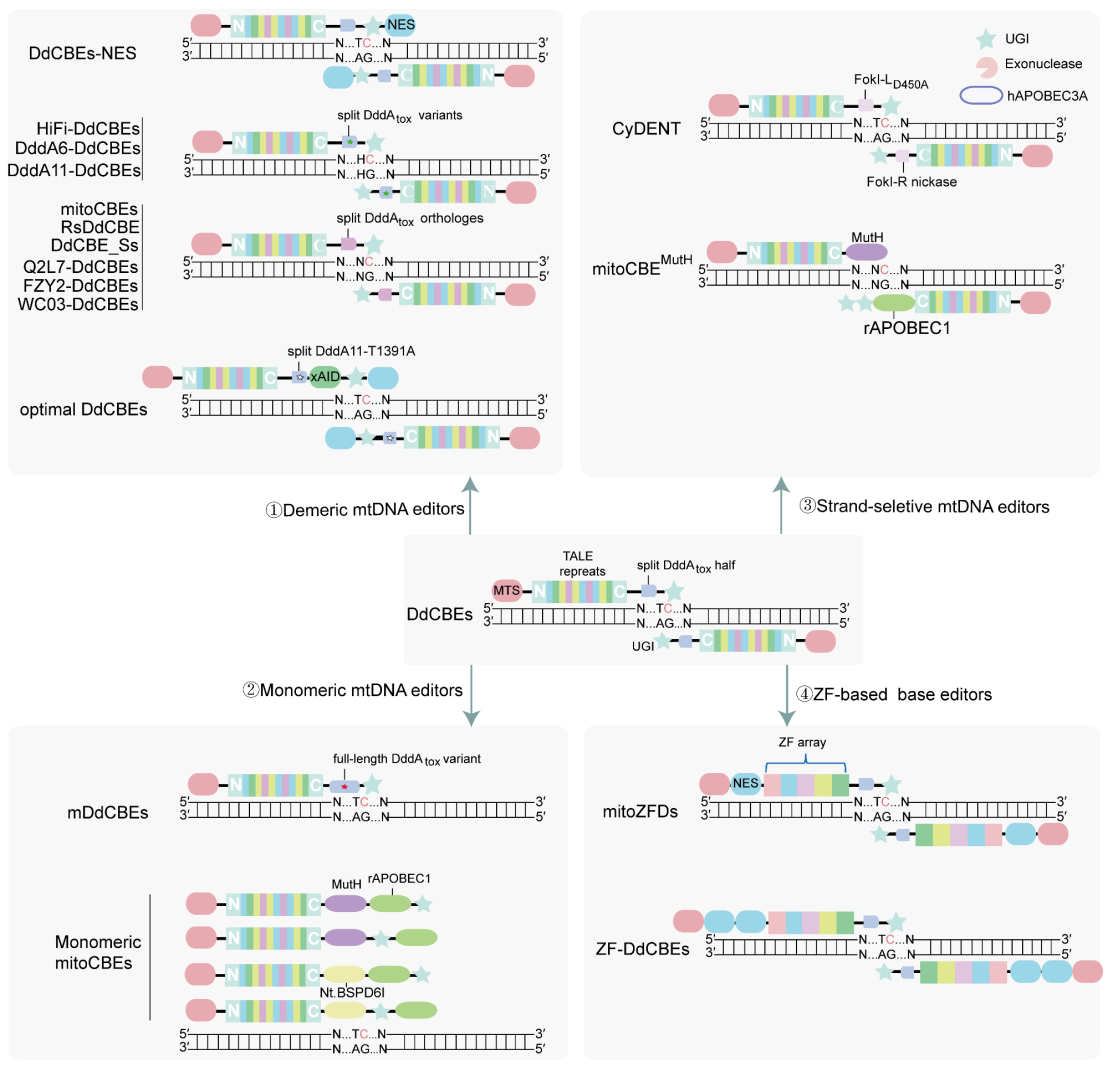
The mitochondrial cytosine base editing toolbox. Target cytosines (C) are shown in red. The originally DdCBEs are a dimer structure consisting of TALE arrays, an UGI, MTS and DddA_tox_ (G1333- or G1397-split). ①Demeric mtDNA editors. The originally DdCBEs have strict TC restriction. Additional NES was fused to DdCBEs, which improved mtDNA editing efficiency at TC targets. Also, DddA_tox_ variants can be used for enhanced activity and broaden editing scope. High-fidelity DddA-derived cytosine base editors (HiFi-DdCBEs) in which DddA_tox_ variants containing K1389A, T1391A, V1411A and T1413A mutations (show in ), and variants DddA6 (show in ) can be used for enhanced activity at TC targets, respectively. Additionally, variant DddA11 (show in ) can be used for C-to-T editing at HC targets (H = A, C or T). Moreover, DddA orthologs can be used for further broaden editing scope. DddA_tox_ homolog from *Roseburia intestinalis* (termed ri DddA_tox_) and *Ruminococcus* sp. *AF17-6* (RsDddA) were used to generated TALE-based mitoCBEs and RsDddA-derived cytosine base editors (RsDdCBE), which catalyzed C-to-T editing without sequence constraints. Besides, DddA from *Streptomyces* sp. *BK438* (Q2L7) *Lachnospiraceae bacterium sunii NSJ-8* (FZY2), and *Simiaoa sunii* (Ddd_Ss) have strong deaminase activity with GC preference, and DddA of *Ruminococcus* sp. *AF17-6* (WC03) is highly compatible to AC context. In addition, to addressed GC targets, DddA11 with T1391A mutation (DddA11-T1391A) and ssDNA deaminase (xAID) were introduced into DdCBE to generate optimal DdCBEs. ②Monomeric mtDNA editors. The dimeric architectures are difficult for gene delivery via viral vectors with a small cargo size. Therefore, to overcome the limitations, mDdCBEs were developed, which relied on full-length, nontoxic DddA_tox_ variants (DddA_tox_ GSVG/E1347A). In addition, monomeric mitoCBEs were created by introducing nickase MutH or Nt.BspD6I(C) and cytosine deaminase rat apolipoprotein B mRNA editing enzyme catalytic polypeptide-like family protein 1 (rAPOBEC1). ③Strand-seletive mtDNA editors. DdCBEs lack any precision for editing a particular base on one strand of DNA. CyDENT was developed to overcome strand-selective base editing, which comprises a pair of a TALE-fused FokI nickase, a single-strand-specific cytidine deaminase (hAPOBEC3A) and an exonuclease. Additionally, mitoCBE^MutH^ achieves efficient and strand-selective mitochondrial base editing by combination of TALE-MutH and TALE-rAPOBEC1-2×UGI in pairs. ④ZF-based base editors. ZF array is smaller than TALE, thus particularly suitable for packaging in AAV. mitoZFDs and ZF-DdCBEs utilize ZFDs instead of TALEs in TALE-based DdCBEs

**Fig. 3:**
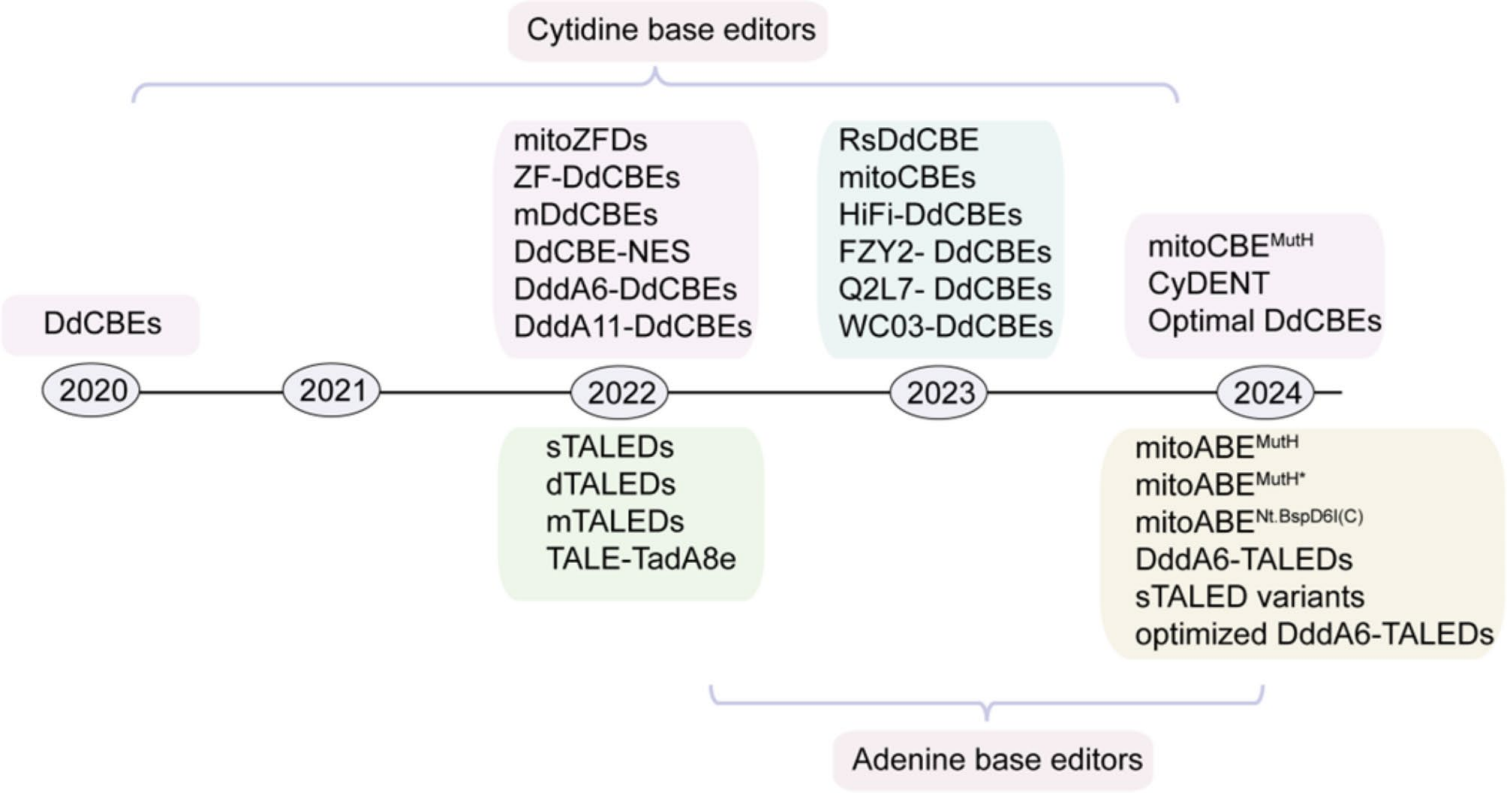
Historical overview of mtDNA editing. The cytidine base editors were initially created in 2020, which catalyze the C-to-T editing within dsDNA without inducing double-strand DNA breaks, making it suitable for mitochondrial genome editing. Several strategies were employed in 2022, including introduction of DddA_tox_ variants, ZFDs and monomeric structure, to expand the editing scope. Meanwhile, adenine base editors were first reported, which were able to catalyze A-to-G conversion in mtDNA. Moreover, DddA orthologs and MutH nickase were introduced in cytidine base editing in 2023 and 2024, further enhancing activity and broadening editing scope. Noting that CyDENT and mitoCBE^MutH^ achieved efficient and strand-selective mitochondrial base editing. Additionally, similar to cytidine base editors, adenine base editors were optimized in 2024 with introduction of DddA_tox_ variants, MutH nickase and Nt.BspD6I(C), increasing activity and wide editing scope

Initially, the David R. Liu group found the interbacterial toxin DddA in *Burkholderia cenocepacia*, which catalyzes the deamination of cytidines within dsDNA without inducing double-strand DNA breaks, making it suitable for mitochondrial genome editing [25, 29]. However, intact DddA_tox_ is toxic to mammalian cells [25]. To solve this problem, DddA_tox_ was split into two inactive halves (DddA_tox_-N and DddA_tox_-C) and fused to TALE array proteins, a UGI and MTS to achieve CRISPR-free base editing in human mtDNA with typical efficiencies ranging between 5% and 50% (Table 1; Figs. 2 and 3) [25]. Moreover, to minimize off-target C-to-T conversions, DdCBEs were fused with an additional nuclear export signal (NES, Table 1; Figs. 2 and 3), enhancing mtDNA editing efficiency by 38.9% and reducing off-target C-to-T conversions in the nuclear genome [30].

To further refine the specificity and efficiency of DdCBEs, Kim et al. engineered high-fidelity DddA-derived cytosine base editors (HiFi-DdCBEs, Table 1; Figs. 2 and 3) by replacing amino acid residues at the interface between the split DddA_tox_ halves with alanine, disrupting the formation of functional deaminase in the absence of TALE-DNA interactions [31]. Specifically, DddA_tox_ variants containing K1389A, T1391A, V1411A, and T1413A mutations are highly active when combined with the wild-type DdCBE, but have low activity with the TALE-free array [31]. Unlike conventional DdCBEs, the HiFi-DdCBEs show highly efficient and precise single-base editing of mtDNA, avoiding collateral off-target mutations [31]. In addition, this variant showed a preference for edited C8 over C9, C11, and C13, whereas the wild-type DdCBEs were promiscuous, editing all four cytosines with high efficiencies of > 19% [31]. The pair containing V1411A in 1397 C was preferentially edited for C11 [31]. Therefore, these compounds may be desirable for therapeutic applications.

However, these DdCBEs are predominantly limited to TC targets due to the strict sequence preference of DddA_tox_. To this end, Mok et al. evolved DddA variants with enhanced editing efficiency and expanded the targeting scope. Phage-assisted continuous evolution (PACE) [32] and phage-assisted non-continuous evolution (PANCE) [33] are methods used to generate improved biomolecules [34]. First, they performed PANCE and obtained a DddA_tox_ (T1380I) mutant (referred to as DddA1) with an average increase in editing efficiency of 1.2-fold to 2.0-fold across the target sites. PACE was then performed to further evolve DddA1 and obtain the variants DddA2 through DddA5 [34]. These variants, particularly DddA5, show improved base editing efficiency. Subsequently, because mutation T1413I from DddA4 might promote reconstitution of split DddA_tox_ halves, they incorporated this variation into DddA5 to form DddA6(Q1310R + S1330I + T1380I + T1413I, Table 1; Figs. 2 and 3), resulting in improved base editing frequencies in TC contexts by 3.3-fold on average, compared to wild-type DddA [34]. Next, to overcome the strict TC sequence-context constraint of DddA, a round of mutagenic drift [35] was conducted, and context-specific PANCE and PACE were performed to generate five DddA variants (DddA7 through DddA11) [34]. Among these variants, DddA11-containing DdCBEs (Table 1; Figs. 2 and 3) offered broadened sequence compatibility at HC (H = A, C, or T) targets and significantly improved mtDNA editing efficiency [34]. However, it remains relatively unavailable for GC targets. Recently, several studies successfully developed DddA orthology-based cytosine base editors that can thoroughly resolve strict sequence-context constraints. There are two methods used to solve this problem: finding new deaminase orthologs and fusing existing DddA variants. The DddA_tox_ homologs from *Roseburia intestinalis* (ri DddA_tox_) and *Ruminococcus* sp. *AF17-6* (RsDddA) were identified and respectively use to generate TALE-based mitochondrial CBEs (mitoCBEs, Table 1; Figs. 2 and 3) and RsDddA-derived cytosine base editors (RsDdCBE, Table 1; Figs. 2 and 3), which catalyzed C-T without sequence constraints in mitochondrial genes [36, 37]. Intriguingly, transactivator (VP64, P65, or Rta) fusion to the UGI of mitoCBEs markedly improved mtDNA editing efficiencies by up to 1.7-fold [37]. Moreover, DddAs from *Streptomyces* sp. *BK438*(Q2L7), *Lachnospiraceae bacterium sunii NSJ-8* (FZY2), and *Simiaoa sunii* (Ddd_Ss) had strong deaminase activity with GC preference, and DddA of *Ruminococcus* sp. *AF17-6* (WC03) is highly compatible with AC context (Table 1; Figs. 2 and 3) [38, 39]. Notably, different split sites affected the off-target activity and context preference of DdCBEs: the Q2L7-DdCBE pairs with T2113 split showed high AC editing activity (up to 34.04%), while the pairs with G2176 displayed strong deaminase activity within the GC context (up to 47.55%) [38]. Additionally, co-expressing the corresponding nuclear-localized DddI_A_ could minimize nuclear off-target editing [38]. Unlike the strategies used in these two studies, a recent study addressed GC targets by fusing the single-stranded activity of cytosine deaminase with DddA11. The fusion of single-stranded DNA (ssDNA) deaminase (xAID) with DddA in an optimal DdCBE variant (DddA11-T1391A-xAID-NES2, Table 1; Figs. 2 and 3) could enhance the editing efficiency and broaden the targeting scope [40].

**Table 1:** List of major mtDNA cytidine base editors with their preferences and efficiency

Base editors	Nucleases	Deaminases and sources	DddA variants	Preferences	Efficiency	References
DdCBEs	TALENs	DddA, *Burkholderia cenocepacia*	-	T*C*	~ 5-50%	[25]
DdCBE-NES	TALENs	DddA, *Burkholderia cenocepacia*	-	T*C*	~ 38.9%	[30]
HiFi-DdCBEs	TALENs	DddA, *Burkholderia cenocepacia*	K1389A, T1391A, V1411A, T1413A	T*C*	~ 59.0%±1.1%	[31]
DddA6-DdCBEs	TALENs	DddA, *Burkholderia cenocepacia*	Q1310R, S1330I, T1380I, T1413I	T*C*	~ 26 ± 3.7%	[34]
DddA11-DdCBEs	TALENs	DddA, *Burkholderia cenocepacia*	S1330I, A1341V, N1342S, E1370K, T1380I, T1413I	H*C* (H = A, C, T)	A*C*(4.3-5.0%) C*C*(7.6–16%) T*C*(~ 20%)	[34]
mitoCBEs	TALENs	DddA, *Roseburia intestinalis*	-	N*C* (H = A, C, G, T)	~ 50%	[37]
RsDdCBE	TALENs	DddA, *Ruminococcus* sp. *AF17-6*	-	N*C*	A*C*(~ 71.51%) C*C*(~ 42.61%) G*C*(~ 86.10%) T*C*(~ 51.28%)	[36]
FZY2- DdCBEs	TALENs	DddA, *Lachnospiraceae bacterium sunii NSJ-8*	-	G*C*	~ 42.55%	[38]
Q2L7- DdCBEs	TALENs	DddA, *Streptomyces* sp. *BK438*	-	G*C*	~ 47%	[38]
				A*C*	~ 34%	
WC03-DdCBEs	TALENs	DddA, *Ruminococcus* sp. *AF17-6*	-	A*C*	~ 38%	[38]
Optimal DdCBEs	TALENs	DddA, *Burkholderia cenocepacia*	DddA11 T1391A	G*C* T*C* C*C*	~ 20-80%	[40]
mDdCBEs	TALENs	DddA, *Burkholderia cenocepacia*	S1326G G1348S A1398V S1418G	T*C*	~ 50%	[41]
CyDENT	TALENs	hAPOBEC3A, human	-	G*C*	~ 14%	[42]
mitoCBE^MutH^	TALENs	rAPOBEC1, rat	-	strand preference	~ 30%	[28]
mitoZFDs	ZFNs	DddA, *Burkholderia cenocepacia*	-	T*C* A*C* GC*C*	~ 30%	[26]
ZF-DdCBEs	ZFNs	DddA, *Burkholderia cenocepacia*	-	T*C*	~ 40%	[43]

Although DdCBEs are highly versatile technologies, their dimeric architectures hamper gene delivery via viral vectors with small cargo sizes. To overcome these limitations, monomeric DdCBEs (mDdCBEs, Table 1; Figs. 2 and 3) have been developed, which rely on non-toxic, full-length DddA_tox_ variants with reduced affinity for dsDNA [41]. First, they generated a library of DddA_tox_ variants by error-prone PCR, and discovered a non-toxic, full-length DddA_tox_ variant with four-point modifications (S1326G, G1348S, A1398V, S1418G), termed the DddA_tox_ GSVG variant [41]. Subsequently, this GSVG variant was fused to the C-terminus of TALE arrays, resulting in mDdCBEs with high frequencies of up to 50% [41]. Notably, the ND4-specific mDdCBE delivered via AAV-induced base editing reached efficiencies as high as 99.1%, demonstrating that mDdCBEs could be packaged into AAV with limited cargo space, facilitating in vivo studies and gene therapy [41]. These results also showed that nearly homoplasmic mutations (> 99%) can be obtained in mtDNA via AAV-mediated base editing without drug selection [41]. Additionally, mDdCBEs can only edit cytoines in TC contexts, and their editing window is positioned 4–11 nucleotides downstream of the TALE-binding site [41]. However, mDdCBEs are more prone to induce mtDNA-wide off-target editing than dimeric DdCBEs. Fortunately, using mDdCBE-encoding mRNAs instead of plasmid DNA was able to reduce or avoid mitochondrial genome-wide off-target effects [41]. MutH is a nickase encoded by *E. coli* that participates in initiating mismatch repair to remove nucleotides misincorporated by the DNA polymerase [44, 45]. Previous studies have achieved DNA nicking by fusing TALE and MutH [46]. Therefore, monomeric mitoCBEs (mitoCBE^MutH^ and mitoCBE^Nt.BspD6I(C)^, Figs. 2 and 3) were developed by introducing nickase MutH or naturally existing nickase Nt.BspD6I(C) [47] and cytosine deaminase rAPOBEC1 [28, 48]. However, wild-type MutH endonuclease activity specifically acts on d(5′-GATC-3′) sequences, limiting the editing scope of monomeric mitoCBEs [49].

Although both DdCBEs can perform efficient C•G-to-T•A base conversions in mtDNA, these editors lack precision for editing a particular base on one strand of DNA. To partially eliminate one of the paired dsDNA molecules at a target site of interest, Hu et al. developed a modular base editing system, cytidine deaminase-exonuclease-nickase-TALE (CyDENT, Table 1; Figs. 2 and 3), a CRISPR-free, strand-selective mtDNA editor [42]. This system comprises a pair of TALE-fused FokI nickase, a single-strand-specific cytidine deaminase Sdd7 [50], and an exonuclease [51–53] to generate a ssDNA substrate for deamination. Wild-type FokI is a bipartite restriction endonuclease, and its recognition domain recognizes a non-palindromic DNA sequence (GGATG (9/13)). Its endonuclease domain cuts the dsDNA outside of the recognition sequence [54–57]. This system preferentially edits GC motifs and shows editing efficiencies of 14% and strand specificity of 95% at mitochondrial sites [42]. rAPOBEC1 is a cytosine deaminase that converts C to T in ssDNA [48]. Previous studies have applied it to the base editing of genomic DNA. Hence, Yi et al. introduced rAPOBEC1 with nickase MutH into strand-selective mtDNA base editing. This editor, named mitoCBE^MutH^ (Table 1; Figs. 2 and 3), achieves mitochondrial C-to-T by combining TALE-MutH and TALE-rAPOBEC1-2×UGI in pairs with a maximum editing efficiency of about ~ 30% [28].

### ZF-based cytidine mtDNA base editors

Compared to TALE arrays (2 × 1.7 ~ 2 kbp) or S. pyogenes Cas9 (4.1 kbp), zinc finger (ZF) arrays (encoded in a 2 × 0.3 ~ 0.6 kbp DNA) are small in such as AAV for studies [58]. Therefore, based on the development of DdCBEs, ZF-based cytidine mtDNA base editors have been engineered to edit mitochondrial DNA in vitro and in vivo. mitoZFDs (Table 1; Figs. 2 and 3) were constructed by which zinc finger deaminases (ZFDs) instead of TALEs in TALE-based DdCBEs [26]. In general, all mitoZFD constructs include MTS, NES, and the N- or C-terminal half of DddA_tox_ split at G1397, a ZF array, and UGI (Fig. 1b) [26]. Because the split architecture of mitoZFDs produced C-type- and N-type arms, there are four possible mitoZFD architectures: CC (left C-type + right C-type), NC (left N-type + right C-type), CN (left C-type + right N-type), and NN (left N-type + right N-type). In nine test target sites in mtDNA, the CC and CN configurations were tested, and mitoZFDs with the CN configuration were observed to have higher editing efficiencies [26]. However, further testing is needed to determine whether the observations are generalizable. Moreover, these mitoZFDs edited cytosines in TC and TCC contexts. Interestingly, this study found that two cytosines (C8 and C9) in an ACC context in the MT-ND2 site were significantly edited, suggesting that mitoZFD is not limited to the TC motif [26].

Similar to mitoZFDs, Willis et al. engineered zinc finger DdCBEs (ZF-DdCBEs, Table 1; Figs. 2 and 3), in which ZF arrays, instead of TALEs, are DNA-binding moieties [43]. Unlike mitoZFDs, which were optimized by varying the length of the amino-acid linker between the ZF arrays and split-DddA_tox_ halves, testing different spacer lengths, and domain ordering, the optimization of ZF-DdCBEs was more comprehensive, including engineering their architectures, defining improved ZF scaffolds, and installing DddA activity-enhancing mutations [26, 43]. Finally, the optimized ZF-DdCBEs possessed improved DNA specificity and a lower off-target effect by integrating these four strategies.

## Creation and optimization of base editors of A•T-to-G•C in mitochondrial DNA

mtDNA cytidine editors exclusively achieve C•G-to-T•A conversion in mtDNA, largely limited to mitochondrial base editing. Based on DdCBE technology, Kim et al. developed TALEDs with the ability to introduce targeted A-to-G conversions in human mtDNA [27]. Similar to DdCBEs, adenine base editors generally have four fundamental components: (i) an N-terminal MTS; (ii) a custom-designed TALE array to target a specific DNA sequence; (iii) a catalytically deficient, full-length DddA variant or split DddA_tox_ (E1347A) originating from *Burkholderia cenocepacia*; (iv) TadA8e, a deoxyadenosine deaminase variant derived from *E. coli* TadA, which catalyzes the hydrolytic deamination of adenine to yield inosine, thus inducing A-to-G conversions (Fig. 1c) [27, 59].

**Fig. 4:**
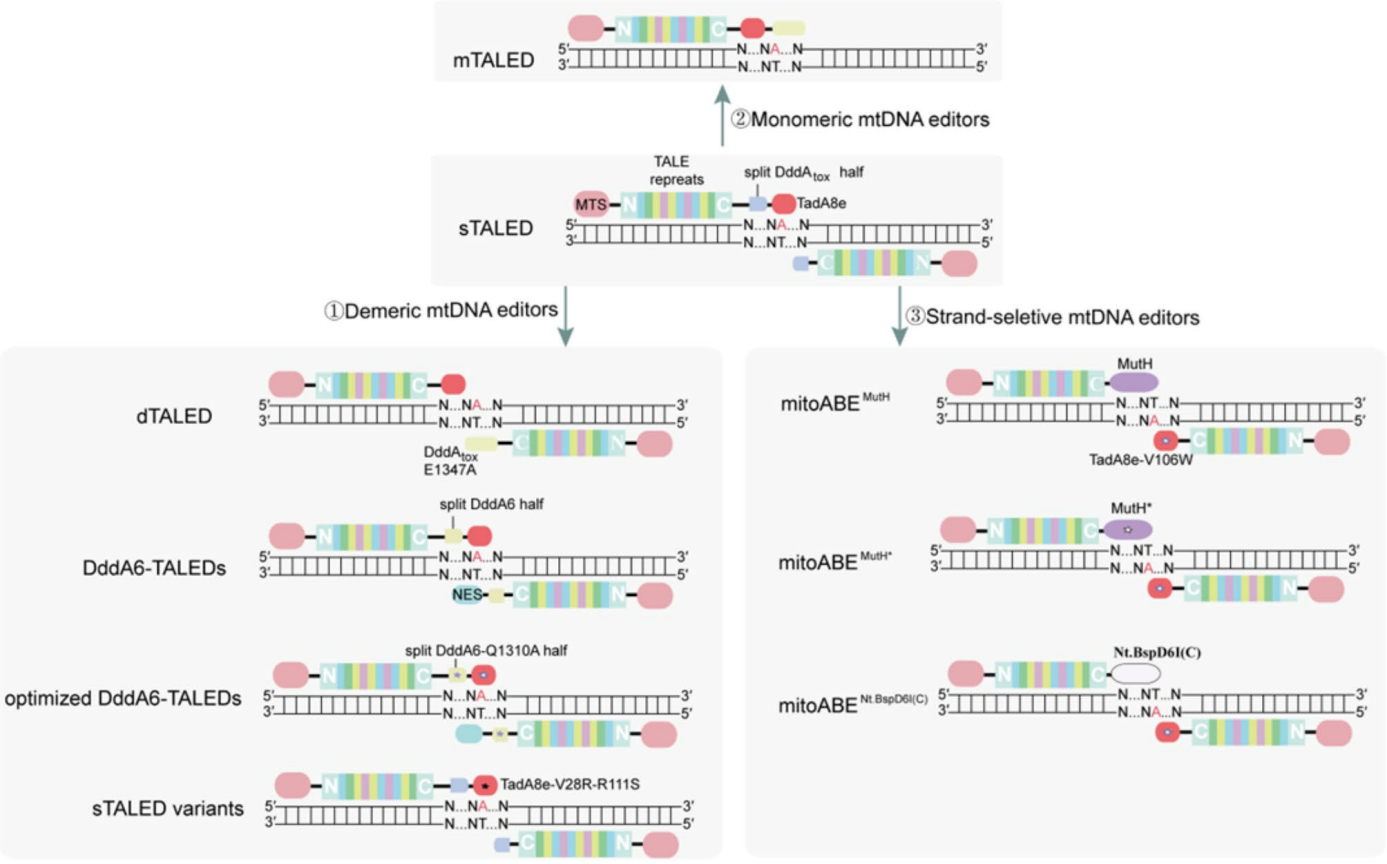
The mitochondrial adenine base editing toolbox. Target adenines (A) are shown in red. The originally sTALED is a dimer structure, comprise TALE arrays, an adenine deaminase TadA8e, a MTS and a split DddA_tox_. ①demeric mtDNA editors. dimeric TALED (dTALED) composed of two TALE arrays in a tail-to-tail configuration to target a desired mtDNA sequence, each fused either to the TadA8e or the E1347A DddA_tox_ variant. Next, DddA6 introduced into TALEDs to create DddA6-TALEDs, and added NES sequences. Moreover, optimized version of TALEDs TadA8e were developed by V106W substitution in TadA8e (show in ), interface mutation (Q1310A) in DddA6 (show in ). Furthermore, sTALED variants are further optimized by introduced site-specific mutations (V28R and R111S, show in ) in TadA8e. ②Monomeric mtDNA editors (mTALED). mTALEDs are composed of a single TALE protein fused to the TadA8e and the full-length DddA_tox_ E1347A, which suitable for viral vectors with a small cargo size. ③Strand-seletive mtDNA editors. mitoABE^MutH^ comprises TALE-MutH and TALE-TadA8e-V106W in pairs. Then, mitoABE^MutH*^ was created by introducing E91A and F94A mutations to MutH (show in ). In addition, Nt.BspD6I nickase was introduced to develop the editing tool named mitoABE^Nt.BspD6I(C)^

Initially, they created the TALE-TadA8e fusion protein (Table 2), and then tested it in the MT-ND1 and MT-ND4 loci in human embryonic kidney 293T (HEK 293T) cells [27]. The results showed poor frequencies, ranging from 0.7–1.2% [27]. In previous studies, TadA variants operate on ssDNA with remarkable efficiency [22, 27, 60]. It was hypothesized that DddA_tox_ operates on dsDNA, providing an accessible ssDNA substrate to the TadA variant [27]. DddA_tox_ was utilized in mtDNA adenine editors to improve the editing efficiency. Similar to the canonical DdCBE architecture, they created split TALE deaminase (sTALED, Table 2; Figs. 3 and 4) by fusing TadA-derived deoxyadenosine deaminase with a TALE protein and split DddAtox (split at G1397 or G1333) [27]. The system catalyzes A-to-G with significant efficiency in mtDNA [27]. Note that keeping a UGI fused to the sTALED arm without the TadA8e enzyme could simultaneously catalyze A-to-G and C-to-T edits in human mtDNA [27]. Two types of TALEDs containing the E1347A DddA_tox_ variant were developed: dimeric and monomeric TALED (dTALED and mTALED, Table 2; Figs. 3 and 4) [27]. The dTALED architecture was similar to that of sTALEDs, composed of two TALE arrays in a tail-to-tail configuration to target a desired mtDNA sequence, each fused either to the TadA8e or the E1347A DddA_tox_ [27]. Whereas mTALEDs were composed of a single TALE protein fused to TadA8e and full-length DddA_tox_ E1347A, resulting in the MTS-TALE-TadA8e-DddA_tox_ E1347A architecture [27]. Both mTALEDs and dTALEDs show significant A-to-G edits with high product purity [27]. Moreover, they compared the editing efficiencies of three types of TALEDs at 12 different sites in human mtDNA [27]. Overall, sTALEDs performed the highest editing frequency of 27%±3%, while mTALEDs and dTALEDs were less active than sTALEDs, with an average A-to-G editing frequency of 19%±4% [27]. Nonetheless, mTALEDs and dTALEDs were more efficient than the corresponding sTALED at specific target sites [27]. Notably, they also found that TALEDs, unlike DdCBEs, can catalyze base editing at sites without the requirement for the 5′-TC motif at a target site [27]. In addition, they targeted the same spacer region with distinct editing patterns in which the editing windows of sTALEDs were positions 5 to 12 and mTALEDs and dTALEDs were positions 7 to 12, widening the scope of mitochondrial genome editing [27]. Considering that DddA variants (DddA6 and DddA11) were introduced into DdCBEs, the activity was improved and the targeting range was expanded. Therefore, DddA6 or DddA11 was introduced into TALEDs to create DddA6-TALEDs (Table 2; Figs. 3 and 4) and DddA11-TALEDs with potentially enhanced editing activity [40]. While DddA11-TALEDs were slightly less efficient than the original TALEDs, DddA6-TALEDs displayed distinctly higher editing efficiencies [40]. Subsequently, Wei et al. developed an optimized version of TALEDs (6CN-Q1310A-AD-V106W-NES1, Table 2; Figs. 3 and 4) with enhanced A-to-G editing efficiency by V106W substitution in TadA8e, interface mutation (Q1310A) in DddA6, and added NES sequences [40].

**Table 2:** List of major mtDNA adenine base editors with their preferences and efficiency

Base editors	Nucleases	Deaminases and sources	TadA8e variants	DddA variants	Efficiency	References
TALE-TadA8e	TALENs	TadA8e, *E. coli*	-	-	0.7-1.2%	[27]
sTALEDs	TALENs	TadA8e, *E. coli*	-	E1347A	27%±3%	[27]
dTALEDs	TALENs	TadA8e, *E. coli*	-	E1347A	19%±4%	[27]
mTALEDs	TALENs	TadA8e, *E. coli*	-	E1347A	19%±4%	[27]
DddA6-TALEDs	TALENs	TadA8e, *E. coli*	-	Q1310R S1330I T1380I T1413I	3-51%	[40]
optimized DddA6-TALEDs	TALENs	TadA8e, *E. coli*	V106W	Q1310A S1330I T1380I T1413I	10-75%	[40]
sTALED variants	TALENs	TadA8e, *E. coli*	V28R R111S	-	at least 50%	[61]
mitoABE^MutH^	TALENs	TadA8e, *E. coli*	V106W	-	At most 100%	[28]
mitoABE^MutH*^	TALENs	TadA8e, *E. coli*	V106W	-	~ 50%	[28]
mitoABE^Nt.BspD6I(C)^	TALENs	TadA8e, *E. coli*	V106W	-	~ 50%	[28]

Whole-transcriptome sequencing revealed that adenine base-edited TALEDs caused tens of thousands of transcriptome-wide off-target A-to-I conversions [61]. To avoid or minimize unwanted editing, Cho et al. introduced site-specific mutations (V106W, V106G, K20A/R21A (dual mutations), or F148A) in TadA8e to create sTALED variants [61]. Although these variants significantly reduce off-target A-to-G edits, they are not complete. To further optimized this system, they engineered TALEDs by mutating amino acid residues and obtained 209 sTALED variants [61]. A total of 101 sTALED pairs showed high base editing efficiency (at least 50%) in the targeted mtDNA [61]. Finally, 12 sTALED variants with minimized RNA off-target editing and retention of mtDNA on-target editing efficiency were obtained. Among them, two TadA8e variants, V28R and R111S, showed higher on-target editing activity and lower RNA off-target activity and bystander edits (Table 2; Figs. 3 and 4) [61]. More importantly, these variants were not cytotoxic and did not cause embryonic developmental arrest in mice [61].

Although the above-mentioned adenine base editors perform base deamination on both strands of dsDNA within the editing window, they lack any precision for editing a particular base on one strand of DNA. MutH is a nickase encoded by *E. coli* that participates in initiating mismatch repair to remove nucleotides misincorporated by the DNA polymerase [44]. Previous studies have achieved strand-specific nicking of DNA by fusing TALE and MutH [46]. Therefore, Yi et al. introduced TALE-MutH and TALE-TadA8e-V106W in pairs to create mitoABE^MutH^ (Table 2; Figs. 3 and 4), which enables targeted strand-biased editing of mtDNA [28]. However, wild-type MutH sequences, limiting the editing scope of mitoABE^MutH^. Hence, E91A and F94A MutH* were designated to expanded targeting scope. The resulting mitoABE^MutH*^ (Table 2; Figs. 3 and 4) editor can be extended to the activity on d (5’-GATN-3’) sequences. Next, to thoroughly overcome sequence-context constraints, Nt.BspD6I nickase was used to develop an editing tool named mitoABE^Nt.BspD6I(C)^ (Table 2; Figs. 3 and 4) [28]. This editor has the precision for single-stranded DNA-specific base editing on mtDNA with no sequence-context constraints.

## Overview of mitochondrial diseases

MDs are some of the most common heritable diseases, generally caused by pathogenic mutations in either nDNA or mtDNA [6, 62]. Epidemiological evidence suggests it has prevalence of ∼1 in 4300 in adults and ∼1 in 6700 in childhood [63, 64]. Point mutations in mitochondrial encoded genes, especially corresponding to subunits of the electron transport chain, as well as in the genes encoding mitochondrial tRNAs and rRNAs, may trigger for MDs [63, 65].

MDs often present with extremely heterogeneous genetic conditions, meaning that both the pathogenic and wild-type mtDNA molecules coexist within the same cell or tissue, and once the concentration of harmful mtDNA variants reaches a critical level of heteroplasmy, there can be biochemical deficiency that can lead to disease [6, 66]. Consistent with the central importance of mitochondrial energy metabolism, defects in mitochondria can affect any tissue and any organ. Particularly, organs with high energy requirements, such as brain, cardiac and skeletal musculature, are particularly vulnerable to the pathogenic mtDNA variants with high concentration of heteroplasm [67].

Heteroplasmic point mutations can cause a variety of clinical symptoms, such as Leber’s hereditary optic neuropathy (LHON) [68], mitochondrial encephalomyopathy with lactic acidosis and strokelike episodes (MELAS) [69], Leigh’s syndrome (LS), neurogenic weakness, ataxia and retinitis pigmentosa [70], and myoclonic epilepsy with ragged red fibers (MERRF) [71, 72]. Disorders can occur at any age, and manifest with any form of symptom, from severe early-onset syndromes to milder late-onset conditions, and ultimately affect specific areas of certain tissues in diverse manners.

Currently, Primary MDs are incurable, which it often gives rise to significant illness and can lead to premature death [64, 73]. Additionally, accumulation of mutations mtDNA also present in age-related multifactorial diseases, including neurodegenerative disease, optic atrophy, deafness and cancer. Therefore, with increasing prevalence of these conditions in our aging population, it is urgent to develop novel approaches to the investigation and prevention or treatment of these diseases. Recent years, Precision medicine is emerging, quietly suitable for considerable sophistication and individual-level tailoring. Gene therapy can directly correct pathogenic mutations in the nuclear genome in cells or tissues [74, 75]. Mitochondrial editing technologies are relatively new method that may become a desire tool to treat MDs, which will drive heteroplasmic state toward a healthy, wild-type mtDNA population [25, 27].

## Applications of mitochondrial base editing

**Fig. 5:**
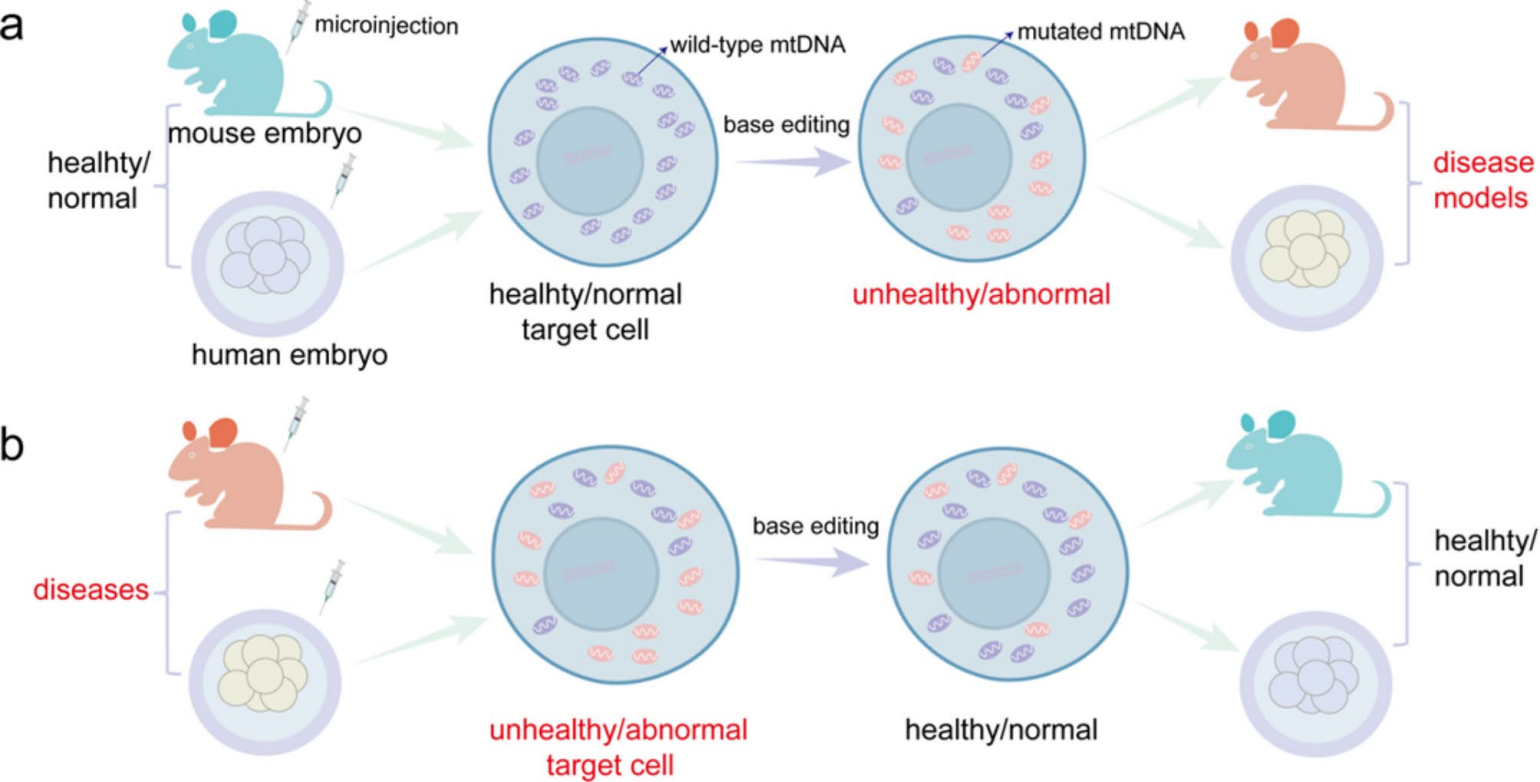
Applications of mitochondrial base editing in mouse models and human embryos. **(a)**Generate mitochondrial disease models by mtDNA editing. mtDNA base editing systems can be designed and microinjection into healthy or normal mouse or human embryos, and which then act on mtDNA molecules to perform specific base editing, driving a wild-type homogeneous condition toward unhealthy or abnormal mtDNA population, thereby obtaining corresponding disease models. **(b)** Elimination of mtDNA Mutations with mtDNA base editors. mtDNA base editors are designed and microinjection into mouse or human embryos with point mutated mitochondrial diseases, and then act on target mutate mtDNA molecules to drive a heteroplasmic condition toward a healthy, wild-type mtDNA population, thereby realize the achieving a cure for the disease

Currently, mitochondrial base-editing systems have achieved C•G-to-T•A and A•T-to- G•C base editing in mtDNA, respectively. Thus, these systems are ideally suited to install and eliminate human pathogenic mtDNA mutation in mouse or human embryos, exploring the physiopathology and develop therapeutic approaches for these diseases. Excitingly, numerous studies have applied mitochondrial base-editing systems to murine models and human embryos, showing the bright promise of mtDNA base editors (Fig. 5).

### Generate mitochondrial disease models by mtDNA editing

MDs are a large class of human hereditary diseases, for which the creation of animal models is essential for understanding the molecular mechanisms underlying mitochondrial disease progression and for developing new treatments. The advent of mtDNA base editors has provided a valuable tool for single-nucleotide modifications in mouse mtDNA. This section focuses on recent studies using mitochondrial base editing techniques in animal models of mitochondrial diseases.

MT-ND5 (ND5) encodes a subunit of NADH dehydrogenase that catalyzes NADH dehydration and electron transfer to ubiquinone. Mutations in the ND5 gene in humans are associated with multiple MDs, including MELAS, as well as some symptoms of LS and LHON [76]. The pathogenic point mutation introduced in the ND5 gene generates mouse models that mimic dysfunctions in humans [77]. First, microinjection of DdCBE-encoding mRNA into mouse embryos introduced m.C12539T and m.G12542A mutations (Table 3). These pathogenic point mutations have been detected in various tissues of newborn (F0) mice, indicating that mtDNA heteroplasmy induced by DdCBEs can be maintained throughout development and differentiation [77]. Then, they crossed the female F0 mouse with a WT male and obtained F1 offspring, and observed that the mutations were also present in F1 offspring, suggesting that mtDNA mutations induced by DdCBEs can be successfully transmitted to the subsequent generation [77]. Meanwhile, Lee et al. created the pathogenic point mutation, m.G12918A mouse model (Table 3), mimicking the m.G13513A variant in human mtDNA [77]. Previous studies confirmed that this mutation in mice leads to multiple mitochondrial diseases, such as LHON, MELAS, and LS [76, 78]. Similarly, Guo et al. utilized DdCBEs to generate mouse models with m.G12918A, m.G7763A, m.G7741A, and m.G2820A mutation, respectively mimicking human mtDNA mutations m.G13513A, m.G8363A, m.G8340A, and m.G3376A. (Table 3) [79]. Human m.G3376A (mouse m.G2820A) and m.G13513A (mouse m.G12918) mutations affect the coding region of MT-ND1 and MT-ND5 genes, respectively, which associated with LHON and MELAS [80]. Additionally, Lee et al. produced the mitochondrial disease models in mice of m.C12336T (Table 3) nonsense mutation incorporating a premature stop codon [77]. Although no apparent phenotypes were observed from these mutations, possibly due to the youth of the animal models or the requirement for a higher level of heteroplasmy for phenotypic translation, this pioneering study demonstrated the potential for creating animal models with mitochondrial dysfunction using mtDNA base editors.

**Table 3:** List of major mtDNA base editing in mouse models

Mouse models	Mimick mutation in human	Locus	Associated Disease	Method	References
m.C12539T	-	MT-ND5	MELAS/LS/LHON	DdCBEs, Install mutation	[77]
m.G12542A	-	MT-ND5	MELAS/LS/LHON	DdCBEs, Install mutation	[77]
m.G12918A	m.G13513A	MT-ND5	Leigh Disease/MELAS/LHON- MELAS Overlap Syndrome/negative association w Carotid Atherosclerosis	DdCBEs, Install mutation	[30, 77, 79]
m.C12336T	-	MT-ND5	MELAS/LS /LHON	DdCBEs, Install mutation	[77]
m.G2820A	m.G3376A	MT-ND1	LHON MELAS overlap	DdCBEs, Install mutation	[79]
m.G7763A	m.G8363A	MT-TK	MICM + DEAF/MERRF/Autism/LS/Ataxia + Lipomas	DdCBEs, Install mutation	[79]
m.G7741A	m.G8340A	MT-TK	Myopathy/Exercise Intolerance/Eye disease + SNHL	DdCBEs, Install mutation	[79]
m.G7755A	m.G8363A	MT-TK	MICM + DEAF/MERRF/Autism/LS/Ataxia + Lipomas	DdCBEs, Install mutation	[81]
m.G14098A	m.G14710A	MT-TE	Encephalomyopathy + Retinopathy	DdCBEs, Install mutation	[81]
m.G007A	m.G583A	MT-TF	MELAS/MM & EXIT	DdCBEs, Install mutation	[82]
m.G1606A	m.G1606A	MT-TV	AMDF	DdCBEs, Install mutation	[82]
m.G11714/5A	m.G12315/6A	MT-TL2	PEO/KSS/possible carotid atherosclerosis risk, trend toward myocardial infarction risk/CPEO	DdCBEs, Install mutation	[82]
m.C5024T	m.G5650A	MT-TA	Myopathy	mitoTALEN, eliminate mutation	[83, 84]

With more in-depth research, a series of studies have observed distinct phenotypes in animal models. Qi et al. applicate DdCBE in rats to generate a mitochondrial disease model of mitochondrial dysfunction [81]. They generated the m.G7755A and m.G14098A rat lines (Table 3) corresponding to human m.G8363A and m.G14710A respectively, associated with mitochondrial disorders [81]. In m.G14098A mutation F1 rats, decreased ATP levels and Complex I activity were observed in the hearts and brains [81]. Subsequently, motor ability was further assessed using an open field test, revealing that G14098A F1 males exhibited decreased movement and average speed, impaired motor coordination and balance, and reduced forelimb grip strength [81]. Furthermore, G14098A mutant rats displayed dilated cardiomyopathy phenotype, characterized by larger chambers and thinner walls, and reduced contraction function as assessed by echocardiography [81]. This study provides important in animal models for studying the mechanisms of mitochondrial disorders and development of clinical therapies. Moreover, Lee et al. also used the DdCBE-NES to generate a mouse model with the m.G12918A mutation in MT-ND5 gene [30]. Mice harboring the m.G12918A mutation displayed a hunchback phenotype, damaged mitochondria in kidney and brown adipose tissues, and abnormal structures in the brain, including aberrantly enlarged ventricles and asymmetrical hippocampal atrophy, ultimately resulting in premature death [30]. Additionally, a study generated the m.G007A, m.G1606A and m.G11714/5A rat models (Table 3) by DdCBE to mimic human pathogenic mutations m.G583A, m.G1606A, and m.G12315/6A, respectively (Table 3) [82]. In human, the m.G583A mutation in mtDNA Trnf (tRNA^Phe)^ is associated with asymptomatic retinopathy and mitochondrial myopathy [85, 86]. The G1606A mutation in mtDNA *Trnv* (tRNA^Val^) are related to a characteristic complex neurological phenotype [87, 88]. The m.G12315/6A mutations in the mtDNA *Trnl2* (tRNA^Leu(CUN)^) gene are linked to atherosclerotic lesions and muscle degeneration-associated syndrome [89, 90]. Among these mutant rats, phenotypes of m.G1030A mutant rats were not observed, probably due to the low mutation loads [82]. m.G007A mutant rats, harboring high mutation loads in toe, skeletal muscle and heart tissues, showed impaired motor ability and a dilated cardiomyopathy (DCM) phenotype [82]. For m.G11714/5A rats, although no motor ability was observed, they exhibited a hypertrophic cardiomyopathy (HCM) phenotype [82]. However, previous studies did not report that patients with G12315/6A mutations could develop HCM. This study might suggest that patients with the G12315/6A dual mutation might be a risk factor of HCM.

Recently, TALEDs were also used to create animal models with pathogenic mtDNA mutations. Researcher obtained animal models by using custom-designed TALEDs targeted two mitochondrial genes, mtATP6 and mtRnr1 [61]. Notably, they obtained mice with m.T8585C and m.T8591C mutations without bystander edits. The two mutations are corresponding to m.T9185C and m.T9191C in the human mtDNA, associated with LS [61]. And these mutant mice showed reduced heart rates (HRs) [61]. Moreover, embryonic fibroblasts (MEFs) of mtATP6 mutant embryo showed significant reduction inoxygen consumption rates, consistent with mitochondrial dysfunction [61]. Taken together, these results showed that TALEDs could be used for creating mouse models with mitochondrial dysfunction.

### Elimination of mtDNA mutations with mtDNA base editors in mouse models

Mitochondria DNA base editors have shown promising to restore mutant mtDNA in mouse model. Recently, AAV9-mitoTALEN was used to restore mutant mtDNA in the muscle of a heteroplasmy mouse model carrying the m.C5024T mutation (Table 3) in the transfer RNA^Ala^ gene [84]. AAV9-mitoTALEN was delivery via intramuscular, intravenous, and intraperitoneal injections, leading to the robust reduction of the mutant mtDNA load both in heart and skeletal muscle [84]. In addition, mitoTALEN also used to selectively eliminate mutant mtDNA within the central nervous system (CNS) of a murine model that carries a heteroplasmic tRNA^Ala^ mutation (m.C5024T) [83]. This mutation is identical to the pathogenic m.G5650A mutation found in human patients with a mitochondrial disease [91, 92]. Moreover, although no overt clinical features were observed in the study, this technology can effectively reduce CNS-mutant mtDNA in vivo, showing promise for clinical trials in patients with mitochondrial encephalopathies [83]. Interestingly, a study expanded the application of DdCBE by incorporating a premature stop codon into the mtDNA-encoding genes to ablate mitochondrial proteins encoded in the mtDNA (mtProteins) instead of installing pathogenic variants or generated a library of cell and rat resources with mtProtein depletion, and then investigated the roles of mtProteins in the heart and brain [93]. This work demonstrates DdCBE could build cell and rat resources for studying the function of mtProtein-coding genes. In conclusion, Mitochondria DNA base-editing systems have potential to restore mutant mtDNA, holding promise for mitochondrial gene therapy.

### mtDNA base editing in human embryos

To evaluate the efficacy and specificity of mtDNA base editors on human early embryo, Chen et al. injected DdCBE mRNAs into the cytoplasm of human Tripronuclear (3PN) zygotes to respectively target three pathogenic mutation sites (G3733A, G8363A, and G13513A), located on human mitochondrial ND1, TRNK, and ND5 genes [94]. The viable 3PN embryos were collected for sequencing on day 3, day 5, and day 6 after microinjected [94]. Previous studies have reported that the mtDNA replication is largely inhibited from the fertilized oocyte stage through the preimplantation embryo stage of human [95]. Due to the DdCBE-mediated base substitution depending on the replication of mtDNA after deamination, the inactive mtDNA replication in the early embryonic stages may affect the editing results. They investigate the status of targeted cytosine in 3PN embryos using dU compatible DNA polymerase (UC) and dU incompatible DNA polymerase (HiFi) [94]. The results showed that up to 37.54% and 58.97% of edits were detected at the G3733A sites, and 44.54% and 10.37% at G8363 site, respectively [94]. However, other bystander mutations within the spacing region were detected with less than 2% editing [94]. These results indicate that DdCBE can mediate mtDNA base conversion efficiently in human 3PN embryos with limited bystander mutations. It’s noted that 255 and 137 off-target sites (OTS) were identified in G3733A and G8363A embryos, respectively [94]. Among these OTS, most of them were detected within 5′-TC motif and 106 OTS were detected in the both edit embryos, suggesting that the off-target editing was mostly caused by DddA_tox_ [94].

To determine the optimal embryonic stage for mtDNA base editing, Wei et al. designed ND4-DdCBE targeting the ND4 locus on human mtDNA [96]. DdCBE mRNAs were injected into human zygote, 2-cell, 4-cell, and 8-cell embryos, respectively, and high editing efficiency was detected after 48 h injection [96]. In particular, human embryos at 8-cell stage showed dramatic increase of the base conversion (up to 60%) with less than 10% cytosine conversion at other stages [96].

Taken together, these two studies first demonstrated that DdCBEs are effective mitochondrial base editors for point mutations in human embryos, and show that DdCBEs are able to correct pathogenic mtDNA mutations in early human embryos, providing a potential treatment for maternal mitochondrial diseases. Although DdCBEs exhibit significant base conversion in human embryo, the existence of bystander and off-target editing limits their application, and further optimization is needed for basic and therapeutic research in the future.

## Challenges of mtDNA base editors

### Bystander and off-target editing with mtDNA base editors

Studies showed that cytidine editing DdCBEs and adenine editing TALEDs not only achieved on-target editing but also induced bystander edits in the editing window, potentially leading to unintended changes [61]. To address this issue, DddA_tox_ variants and its homolog have been evolved to create higher efficient and precise DdCBEs, such as RsDdCBE and HiFi-DdCBE [31, 36]. While these editors can minimize or avoid unwanted bystander edits often observed with DdCBEs, bystander edits may still occurs depending on the target site. In addition, Similar to the DNA base editing technology, mtDNA base editors have the potential to induce off-target editing [97, 98]. Off-target base editing can be classified into “off-target editing within the mtDNA”, which is non-specific editing near the target loci of mtDNA, “off-target editing beyond mtDNA”, which is nuclear genome or RNA off-target edits [97, 98].

Mok and colleagues reported DdCBE can cause low-frequent off-target events on mtDNA and no significant off-target editing at nuclear pseudogenes [25]. However, Wei et al. performed GOTI (genome-wide off-target analysis by two-cell embryo injection) and found DdCBE pairs cause non-specific editing near the target loci of mtDNA [97]. Then, GOTI results demonstrated mitochondrial base editor can produce thousands of off-target single-nucleotide variations (SNVs) enriched for C-to-T/G-to-A base conversion in the entire nuclear genome [97]. Likewise, Lei et al. developed dU-intermediate tracing method Detect-seq, and identified prevalent off-target edits in the nuclear genome, which is induced by DdCBE [98]. However, TALEDs off-target edits were largely limited to A-to-G conversions in mtDNA, TALEDs containing an MTS do not have off-target effects on nDNA at text sites [27]. In addition, adenine editing TALEDs but not cytosine editing DdCBEs induced substantial of transcriptome-wide off-target edits in human cells [61]. Although most of these unwanted RNA off-target edits disappeared several days after transfection, TALEDs were cytotoxic, reducing cell viability leading to developmental arrest of mouse embryos [61].

Notably, DdCBE off-target sites did not overlap with sTALED off-target sites in ND1 test sites, indicating that off-target effects caused by deaminases are independent of the TALE arrays [27]. Interestingly, studies showed most of off-target locs were found in the displacement-loop (D-loop), a highly polymorphic, non-coding region of 1.1kbp in length in human mtDNA [27].

Taking together, scientists need to fully define and characterize mtDNA off-target effects of deaminase enzymes in mtDNA base editor platforms to ensure the safety of gene therapy applications. Further studied need to create advanced mtDNA base editor with high specificity and low cytotoxicity, especially before being used for clinic treating of mitochondrial diseases.

### Target limitations

Due to the original version of cytidine editing DdCBEs have strict TC sequence-context constraint, the target without the TC context cannot be edited [25]. It is speculated that more than 150 loci in the human mitochondrial genome are inaccessible to these base editors. Several attempts had been done to circumvent this constraint, including introducing DddA_tox_ variants and its homolog or developing mDdCBEs or replacing TALEs with DNA-binding domains [36, 41]. Ultimately, the targeting scope of mtDNA cytidine editors were accessible to HC (H = A, C, T). Fortunately, DdCBEs were developed enable efficient, specific, and unconstrained mitochondrial base editing [34, 99]. However, its spacers ranging from 11 to 18 bp long (preferably up to 16 bp), which still cannot precisely target one base with high efficiency [99]. Moreover, these results were obtained from immortalized human cells in vitro, further characterizations and application in clinically relevant cell types and in vivo models are needed to continue to validate [99].

Unlike DdCBEs, adenine editor TALEDs could catalyze A to G base editing without the requirement for the TC motif, which is recognized by DddA_tox_ [27]. Whlie adenine positions 5 to 12 for sTALEDs or 7 to 12 for mTALEDs and dTALEDs were efficiently edited, precisely target one base is still not reached [27]. Besides, editing efficiency depends on the target sites, once with low efficiency even inefficiency, there is still no alternative method.

In conclusion, there are still no perfect mtDNA base editors capable of precisely target single-nucleotide with high efficiency, and not every site can be effectively edited using current editors, which limits its application in gene therapy, in particular, pathogenic single-nucleotide polymorphism. Further studies need to enhanced editors with high compatible with various deaminase domains and eventually access virtually any sequence in human mtDNA.

### Base substitution limitation

The cytidine mtDNA editors and adenine mtDNA editors now can only achieve A•T-to-G•C and G•C-to-A•T conversion in mtDNA. However, among a total of 95 clinically confirmed pathogenic mtDNA mutations, listed in mitomap (www.mitomap.org), there are showed known pathogenic single nucleotide polymorphism (SNPs) caused by other types of base pairs mtDNA mutations such as C•G-to-A•T, A•T-to-C•G, C•G-to-G•C, G•C-to-C•G, A•T-to-T•A, and T•A-to-A•T. Unfortunately, current technology is not able to solve these mutations, so it is urgent to developed new enzymes and systems to expand the application of the mtDNA base-editing system.

### The challenge of accurately modulating mtDNA heteroplasmy

Unlike genetically stable genomic diseases, mitochondrial diseases often occur with a situation known as heteroplasmy, meaning that the mutated and wild-type mtDNA are dynamically coexist in the same cell where the level of mutated mtDNA within the cell is critical as, once it reaches a critical threshold, there can be biochemical deficiency that can lead to disease [6]. Heteroplasmic state is a critical finding in some patients with pathogenic mtDNA variants, and the higher level of mutated mtDNA, the more severe symptoms that a patient is likely to have [100].

mtDNA base editing systems can be designed to specifically act on mutate mtDNA molecules, driving a heteroplasmic condition toward a healthy, wild-type mtDNA population [25, 27]. However, these systems still cannot precisely modulating mtDNA heteroplasmy condtion, meaning that it unreached accurately regulate the ratio of mutate mtDNA and wild-type mtDNA. Particually, considering low base editing efficiencies at some targets, it even disables to drive a heteroplasmic condition toward a healthy, wild-type mtDNA population in cells with a high heteroplasmic load. Moreover, heteroplasmy levels may fluctuate during the cell division (mitotic or meiotic). When the harmful mtDNA are predominantly inherited, it may also cause disease in the next generation. Thus, achieving a therapeutic threshold of wild-type mtDNA using mtDNA single-base editing systems remains a significant challenge.

## Conclusions and future perspectives

Cytidine mtDNA editors and adenine mtDNA editors enable programmable C•G-to-T•A and A•T-to-G•C conversions in mtDNA without requiring dsDNA breaks, which are becoming a novel potential tool to model mitochondrial disease, correct mtDNA pathogenic variants and expand our knowledge of mitochondrial biology.

With the rapid development of mtDNA editing technologies, quite a lot of studies manipulated mitochondrial genetic to resembled human mitochondrial mutations in human cells or rats or zebra fish, which provides important in vitro and vivo models to study the mechanisms of mitochondrial disorders. Moreover, DdCBE can correct pathogenic mtDNA mutations in early human embryos, providing a potential treatment for maternal mitochondrial diseases.

Although mtDNA base editors exhibit efficient, precise, and irreversible base editing, the exist of bystander, off-target editing and base substitution limitation hampers their application. Whether we can more accurately base editing, and further widen the types of base conversion (such as C-to-A or A-to-C), these work remains to be further explored.

In summary, the emergence and development of mitochondrial base editor broaden understanding of mitochondrial diseases. Probably, combined with the continuous development of delivery technology, it will becoming more favorable and promising base editing tool for biomedical research and gene therapy of mitochondrial diseases.

## Data Availability

Data sharing is not applicable to this article as no datasets were generated or analysed during the current study.

## Abbreviations

3PN: Tripronuclear
bp: Base pairs
CNS: Central nervous system
CRISPR: Clustered regularly interspaced short palindromic repeats
CyDENT: Cytidine deaminase-exonuclease-nickase-Tale
DdCBEs: DddA-derived cytosine base editors
D-loop: Displacement-loop
dsDNA: Double-stranded DNA
dTALED: Dimeric TALED
GOTI: Genome-wide off-target analysis by two-cell embryo injection
gRNA: Guide RNA
HCM: Hypertrophic cardiomyopathy
HEK 293T: Human embryonic kidney 293T
HiFi-DdCBEs: High-fidelity DddA-derived cytosine base editors
HRs: Heart rates
LHON: Leber’s hereditary optic neuropathy
LS: Leigh’s syndrome
mDdCBEs: Monomeric DdCBEs
MDs: Mitochondrial diseases
MEFs: Embryonic fibroblasts
MELAS: Mitochondrial encephalomyopathy, lactic acidosis, and stroke-like episodes
MERRF: Myoclonic epilepsy with ragged red fibers
mitoCBEs: Tale-based mitochondrial CBEs
mitoZFDs: Mitochondria-targeting ZFDs
mTALED: Monomeric taled
mtDNA: Mitochondrial DNA
MTS: Mitochondrial targeting sequence
NES: Nuclear export signal
OTS: Off-target sites
PACE: Phage-assisted continuous evolution
PANCE: Phage-assisted non-continuous evolution
SNPs: Single nucleotide polymorphism
SNVs: Single-nucleotide variations
ssDNA: Single-stranded DNA
sTALED: Split tale deaminase
TALEDs: Tale-linked deaminases
TALENs: Transcription activator-like effector nucleases
UGI: Uracil glycosylase inhibitor
ZF: Zinc finger
ZF-DdCBEs: Zinc finger DdCBEs
ZFDs: Zinc finger domain
ZFNs: Zinc-finger nuclease
